# Multiscale Dispersion Entropy of Resting‐State EEG in Older Adults with Alzheimer's Dementia, Mild Cognitive Impairment, or remitted Major Depressive Disorder

**DOI:** 10.1002/alz70856_105953

**Published:** 2026-01-09

**Authors:** Hamed Azami, Mary Pat McAndrews, Mostafa Rostaghi, Reza Zomorrodi, Heather Brooks, Daniel M. Blumberger, Corinne E. Fischer, Alastair Flint, Nathan Herrmann, Sanjeev Kumar, Damien Gallagher, Linda Mah, Benoit H. Mulsant, Bruce G. Pollock, Tarek K Rajji, PACt‐MD Study Group

**Affiliations:** ^1^ Centre for Addiction and Mental Health, Toronto, ON, Canada; ^2^ University of Toronto, Toronto, ON, Canada; ^3^ Toronto Dementia Research Alliance, Toronto, ON, Canada; ^4^ Toronto Western Hospital, University Health Network, Toronto, ON, Canada; ^5^ Department of Mechanical Engineering, Semnan University, Semnan, Semnan, Iran (Islamic Republic of); ^6^ Campbell Family Mental Health Research Institute, Centre for Addiction and Mental Health, Toronto, ON, Canada; ^7^ Temerty Centre for Therapeutic Brain Intervention, CAMH, Toronto, ON, Canada; ^8^ Adult Neurodevelopment and Geriatric Psychiatry Division, Centre for Addiction and Mental Health, Toronto, ON, Canada; ^9^ Department of Psychiatry, Temerty Faculty of Medicine, University of Toronto, Toronto, ON, Canada; ^10^ Temerty Centre for Therapeutic Brain Intervention CAMH, Toronto, ON, Canada; ^11^ Temerty Faculty of Medicine, Department of Psychiatry, University of Toronto, Toronto, ON, Canada; ^12^ Keenan Research Centre for Biomedical Science, Li Ka Shing Knowledge Institute, St. Michael's Hospital, Toronto, ON, Canada; ^13^ University Health Network, Toronto, ON, Canada; ^14^ Temerty Faculty of Medicine, University of Toronto, Toronto, ON, Canada; ^15^ Sunnybrook Health Sciences Centre, Toronto, ON, Canada; ^16^ Department of Psychiatry, University of Toronto, Toronto, ON, Canada; ^17^ Rotman Research Institute, Baycrest Health Science Centre, Toronto, ON, Canada; ^18^ Adult Neurodevelopment and Geriatric Psychiatry Division, CAMH, Toronto, ON, Canada; ^19^ Department of Psychiatry, Temerty Faculty of Medicine, University of Toronto, Toronto, ON, Canada, Toronto, ON, Canada; ^20^ UT Southwestern University, Dallas, TX, USA

## Abstract

**Background:**

Multiscale dispersion entropy (MDE) is a nonlinear approach for assessing the complexity of brain activity using electroencephalograms (EEGs). MDE captures EEG dynamics across biologically relevant time scales, with short‐scales reflecting high‐frequency oscillations and local neuronal activity, and long‐scales representing low‐frequency oscillations and large‐scale network processes. Previous studies suggest that patients with Alzheimer's dementia (AD) have decreased complexity at short time scales compared to those with mild cognitive impairment (MCI) or healthy controls (HCs), and individuals with MCI show reduced complexity compared to HCs. There is also preliminary evidence suggesting that adult patients with acute depression –a high‐risk condition for AD– have decreased complexity at a short time scale. Thus, we conducted a study in older participants with AD, MCI, HC, remitted major depressive disorder (rMDD), or rMDD+MCI, hypothesizing reduced short‐scale MDE in AD vs. MCI and MCI vs. HC. We also explored MDE at short and long time scales across all diagnostic groups and their relationships with cognitive performance.

**Method:**

The study included 44 HC, 46 rMDD, 114 MCI, 71 rMDD+MCI, and 41 AD participants. MDE was generated using resting‐state EEG with 24ms as the short time scale and 60ms as the long time scale. Cognition was assessed using the Montreal Cognitive Assessment and a cognitive composite score from a comprehensive neuropsychological battery.

**Result:**

MDE at 24ms was decreased in AD vs. MCI and in MCI vs. HCs. rMDD had no impact. At 60ms, only the AD group differed from the other groups. Cognitive performance was associated with MDE at 24ms but not 60ms.

**Conclusion:**

This study highlights the value of MDE at a short time scale, related to local neuronal activity, to separate individuals with AD vs. MCI vs. HCs. Reduced complexity in these individuals may underlie their cognitive impairment. In contrast, our study suggests that any MDD impact on complexity is likely related to active depressive symptoms.

